# Epitope-specific antibody responses differentiate COVID-19 outcomes and variants of concern

**DOI:** 10.1172/jci.insight.148855

**Published:** 2021-07-08

**Authors:** Courtney Voss, Sally Esmail, Xuguang Liu, Michael J. Knauer, Suzanne Ackloo, Tomonori Kaneko, Lori Lowes, Peter Stogios, Almagul Seitova, Ashley Hutchinson, Farhad Yusifov, Tatiana Skarina, Elena Evdokimova, Peter Loppnau, Pegah Ghiabi, Taraneh Haijan, Shanshan Zhong, Husam Abdoh, Benjamin D. Hedley, Vipin Bhayana, Claudio M. Martin, Marat Slessarev, Benjamin Chin-Yee, Douglas D. Fraser, Ian Chin-Yee, Shawn S.C. Li

**Affiliations:** 1 Department of Biochemistry and; 2 Department of Pathology and Laboratory Medicine, Schulich School of Medicine & Dentistry, Western University, London, Ontario, Canada.; 3 Structural Genomics Consortium and; 4 Department of Chemical Engineering and Applied Chemistry, University of Toronto, Toronto, Ontario, Canada.; 5 Department of Medicine, Western University, London, Ontario, Canada.; 6 London Health Sciences Centre, London, Ontario, Canada.; 7 Divison of Hematology and; 8 Department of Paediatrics, Schulich School of Medicine & Dentistry, Western University, London, Ontario, Canada.

**Keywords:** COVID-19, Infectious disease, Immunoglobulins

## Abstract

**BACKGROUND:**

The role of humoral immunity in COVID-19 is not fully understood, owing, in large part, to the complexity of antibodies produced in response to the SARS-CoV-2 infection. There is a pressing need for serology tests to assess patient-specific antibody response and predict clinical outcome.

**METHODS:**

Using SARS-CoV-2 proteome and peptide microarrays, we screened 146 COVID-19 patients’ plasma samples to identify antigens and epitopes. This enabled us to develop a master epitope array and an epitope-specific agglutination assay to gauge antibody responses systematically and with high resolution.

**RESULTS:**

We identified linear epitopes from the spike (S) and nucleocapsid (N) proteins and showed that the epitopes enabled higher resolution antibody profiling than the S or N protein antigen. Specifically, we found that antibody responses to the S-811–825, S-881–895, and N-156–170 epitopes negatively or positively correlated with clinical severity or patient survival. Moreover, we found that the P681H and S235F mutations associated with the coronavirus variant of concern B.1.1.7 altered the specificity of the corresponding epitopes.

**CONCLUSION:**

Epitope-resolved antibody testing not only affords a high-resolution alternative to conventional immunoassays to delineate the complex humoral immunity to SARS-CoV-2 and differentiate between neutralizing and non-neutralizing antibodies, but it also may potentially be used to predict clinical outcome. The epitope peptides can be readily modified to detect antibodies against variants of concern in both the peptide array and latex agglutination formats.

**FUNDING:**

Ontario Research Fund (ORF) COVID-19 Rapid Research Fund, Toronto COVID-19 Action Fund, Western University, Lawson Health Research Institute, London Health Sciences Foundation, and Academic Medical Organization of Southwestern Ontario (AMOSO) Innovation Fund.

## Graphical abstract

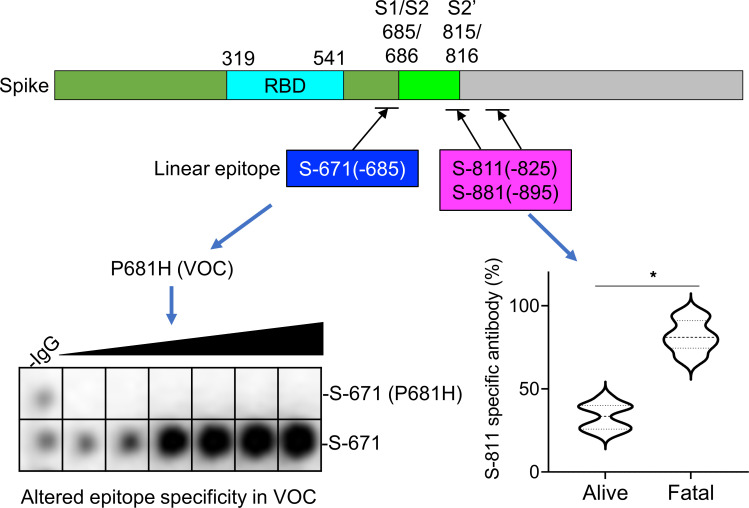

## Introduction

SARS-CoV-2 has infected more than 150 million people worldwide since it was first identified in humans in 2019. The ensuing COVID-19 pandemic has put diagnostic testing at the forefront in the battle to stop the spread of the virus. Nucleic acid testing (NAT), which detects the virus RNA by reverse-transcription polymerase chain reaction (RT-PCR), is the current gold standard for diagnosing acute infections (1). NAT has played a critical role in containing the pandemic by allowing expedient identification of infected individuals for treatment, isolation, and contact tracing. However, NAT alone cannot reveal the true prevalence of the SARS-CoV-2 infection because 20% to 80% of all infections are likely asymptomatic (2–4). Therefore, a significant proportion of the population would be missed by NAT-based screening because the virus is typically cleared by the immune system in 3 to 4 weeks after infection or symptom onset. To complement NAT, serological assays for virus-specific antibodies have been developed (5–7). In contrast to NAT that can only detect acute infections, serology tests can identify past infections because antibodies may persist in the blood long after the virus has been cleared. The wide window of time within which antibodies may be detected, ranging from 1 to 2 weeks of infection when seroconversion occurs to several months after the infection has been resolved, offers a unique advantage for antibody testing over NAT. Because of the high incidence of asymptomatic cases, antibody testing, when carried out in large scale, can provide valuable and accurate information about the spread of the infection at the population level and the true infection fatality rate (8, 9). Importantly, with the advent of several effective vaccines against the virus and the rapid rollout of the vaccination program around the world, priorities are being shifted from containment to monitoring the immediate and longitudinal effects of the vaccines on the immune system. This paradigm shift will undoubtedly increase the demand for antibody testing.

Numerous serological assays for SARS-CoV-2 antibodies have been developed to date, which include enzyme-linked immunosorbent assays (ELISAs), chemiluminescence immunoassays, and lateral flow assays (LFAs; refs. 1, 8). The sensitivity and specificity of different ELISA kits may vary (10), but they are generally considered sufficient for large-scale SARS-CoV-2 antibody testing. Nevertheless, the need for specialized equipment and trained personnel to perform the test and the long turnaround time make it a challenge to use ELISA in point-of-care (POC) settings. In contrast, LFAs, which can be carried out in less than 30 minutes with no equipment required, can potentially be used for POC testing. However, LFA-based tests have been shown to be less sensitive and specific than ELISAs (6, 9, 11, 12). Besides concerns over sensitivity, specificity, and POC potential, both ELISA- and LFA-based antibody testing have the following limitations. First, current tests rely on the interaction of the spike (S) or nucleocapsid (N) protein or a fragment/domain of either protein to capture the corresponding antibody. These assays, which provide a single measure of antibody reactivity, are not ideal for gauging the diverse antibody responses observed in the clinic. Second, protein antigen-based immunoassays such as ELISA and LFA generate a composite signal across many epitopes, including both conformational and linear epitopes, thereby lacking the necessary specificity or resolution to differentiate between neutralizing and non-neutralizing antibodies or predict clinical outcome. Indeed, patients who are older or with severe symptoms have been shown to produce more antibodies than those who are younger or with milder symptoms (13, 14), suggesting that robust antibody responses measured by conventional means do not correlate with effective humoral immunity. Third, current serological assays are ill-suited to assess the immunological effect of coronavirus variants because numerous recombinant proteins would have to be produced. Several mutated strains have emerged recently that are believed to be more contagious than the original SARS-CoV-2 strain (15, 16). These and other variants of concern (VOCs) harbor numerous missense or deletion mutations in the S or N protein-encoding gene that may alter their antigenic characteristics. To effectively curb the spread of these highly contagious VOCs, it is of paramount importance that we develop an antibody test that can readily incorporate the emerging mutations to determine the effect of these mutations and the corresponding VOCs on the immune system. Fourth, current immunoassays are generally focused on testing a specific antibody isotype. Given the distinct dynamics of IgM, IgA, and IgG in response to the SARS-CoV-2 infection (17, 18), it is necessary to develop a multiplex immunoassay to gauge humoral immunity. Last, with the vaccine rollout across the globe, a rapid and accurate POC test is urgently needed to gauge the effectiveness of a vaccine and monitor the duration of antibody responses in large populations to provide reliable information on herd immunity.

We addressed these unmet needs in SARS-CoV-2 antibody testing using protein and peptide arrays, which led to the identification of linear epitopes that mediate the complex antibody responses observed in a group of 89 patients with COVID-19. This, in turn, allowed us to develop a “master epitope array” containing the major epitopes and use it to gauge antibody responses with greater resolution than is attainable by protein antigen-based immunoassays. We found that the antibody profiles determined by linear epitopes, but not by S or N protein, could distinguish patients with moderate or severe diseases or with favorable or fatal outcomes. Using a peptide array recapitulating the mutations found in SARS-CoV-2 variants, we showed that certain mutations abolished binding of the corresponding epitopes to antibodies against the original strain. Furthermore, the identified epitopes enabled us to develop an epitope-dependent agglutination assay for SARS-CoV-2 antibodies. This rapid agglutination assay is not only highly accurate, but it can also be readily modified to incorporate specific epitopes, including VOC epitopes, to profile the complex antibody responses in individuals.

## Results

### Antibody responses to the S and N proteins are not correlated with clinical outcome.

**Figure 1:**
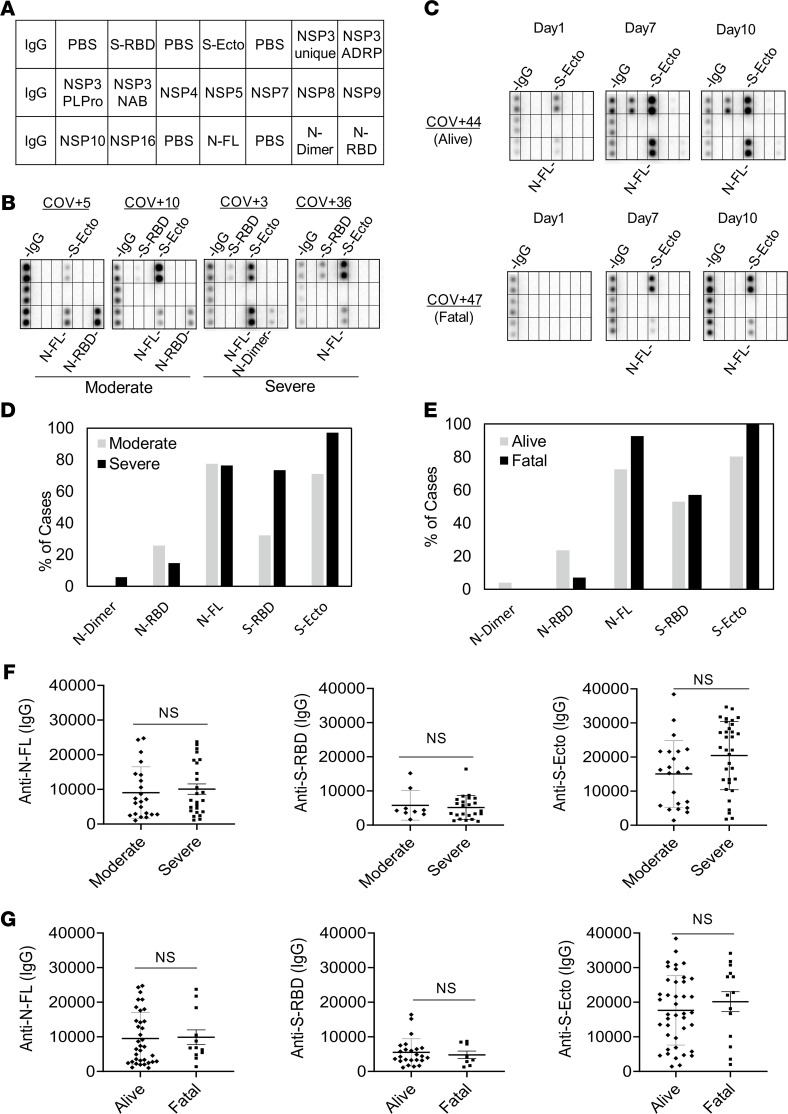
Lack of correlation between the spike or nucleocapsid antibody response and disease severity or outcome. (**A**) Layout of the SARS-CoV-2 proteome array. The array included immunoglobulin G (IgG), phosphate-buffered saline (PBS), spike receptor-binding domain (S-RBD), spike ectodomain (S-ecto), nonstructural protein (NSP), ADP-ribose-1′′-monophosphatase (ADRP), papain-like protease (PLPro), nucleic acid binding (NAB), nucleocapsid full length (N-FL), nucleocapsid dimerization domain (N-dimer), and nucleocapsid RNA-binding domain (N-RBD). (**B**) Representative images (from *n* = 65 unique patient samples) of antibody responses for COVID-19 patients with moderate or severe disease determined by the proteome array. (**C**) Dynamic IgG antibody profiles for 2 patients with severe (but alive) or fatal disease on days 1, 7, and 10 of intensive care unit (ICU) admission. (**D** and **E**) Prevalence of antibody responses to the S or N protein/domain for the indicated patient groups determined by the proteome array (based on high-exposure images). (**F** and **G**) The intensity of antibody response to the S or N protein antigen was not correlated with disease severity (**F**) or outcome (**G**). IgG-binding signals were based on low-exposure array images. Intensity cutoff value was set at 2 SDs of the mean background signal at low exposure. Moderate, *n* = 31; severe, *n* = 34; alive, *n* = 51; fatal *n* = 14. NS, not significant from unpaired Student’s *t* test with Welch’s correction.

To develop a comprehensive antibody test, we first employed a protein array to identify the SARS-CoV-2 antigens mediating antibody responses. Previous studies have implicated the S, N, and nonstructural proteins encoded by the *ORF1ab* gene as the major antigens eliciting humoral immune responses in the host (19, 20). We therefore expressed these proteins, including fragments or domains of S and N, in bacterial or mammalian cells. Upon purification, the recombinant virus proteins were printed on nitrocellulose-coated glass slides. The resulting proteome array, featuring 16 SARS-CoV-2 proteins and human IgG as the positive control (Figure 1A, Supplemental Table 1; supplemental material available online with this article; https://doi.org/10.1172/jci.insight.148855DS1), was probed with plasma samples from patients that tested positive or negative for SARS-CoV-2 by RT-PCR (10). The bound IgG was detected using goat anti–human IgG conjugated to horseradish peroxidase (HRP; Supplemental Figure 1).

We screened the proteome array and subsequent peptide arrays (*vide infra*) with 146 plasma samples from 89 hospitalized patients, including serial samples collected for some patients on different days after diagnosis. The patients were divided into 2 groups with severe (i.e., admitted to the intensive care unit, ICU) or moderate (i.e., no intensive care required) disease. The same patient cohort was also classified according to clinical outcome into the “alive” or “fatal” group, with the former comprising those who survived the infection (consisting of both moderate and severe cases) and the latter who ultimately succumbed to the disease (consisting only of severe cases) (Supplemental Table 2). As shown in Figure 1B, both the moderate and severe groups showed IgG responses to the spike (including the ectodomain, S-ecto, and receptor-binding domain, S-RBD) and the nucleocapsid protein (including the RNA-binding domain, N-RBD, and the dimerization domain, N-dimer). In contrast, no significant IgG-binding signal was detected for the NSP proteins (Figure 1, A–C, and Supplemental Figure 2). These results are consistent with previous findings by others that the spike and nucleocapsid are the main antigenic proteins in SARS-CoV-2 (19–23). For the ICU patients with serial plasma samples, we found that the S/N-specific IgG signals increased from day 1 (of ICU admission) to days 7 and 10 for both the alive and the fatal groups (Figure 1C). This indicates that humoral immune responses became more robust with time in these patients regardless of outcome.

Overall, we found that all seroconverted patients showed IgG responses to either the S or N protein or both. A greater percentage of the severe patient group had antibodies specific for S-RBD or S-ecto than those with moderate conditions. In contrast, the difference in N-specific IgG signal was small between the 2 groups (Figure 1D). Compared with the group that survived the infection, the fatality group more frequently exhibited S- or N-specific antibodies (Figure 1E), suggesting once again that a robust antibody response does not necessarily translate into a favorable outcome. In support of this assertion, we found no correlation between the strength of S- or N-specific IgG signal and disease severity or outcome (Figure 1, F and G). Taken together, the proteome array screen data indicate that the S or N antibody response is not a sensitive barometer of COVID-19 clinical severity or outcome.

### Systematic identification of linear epitopes by peptide microarrays.

**Figure 2:**
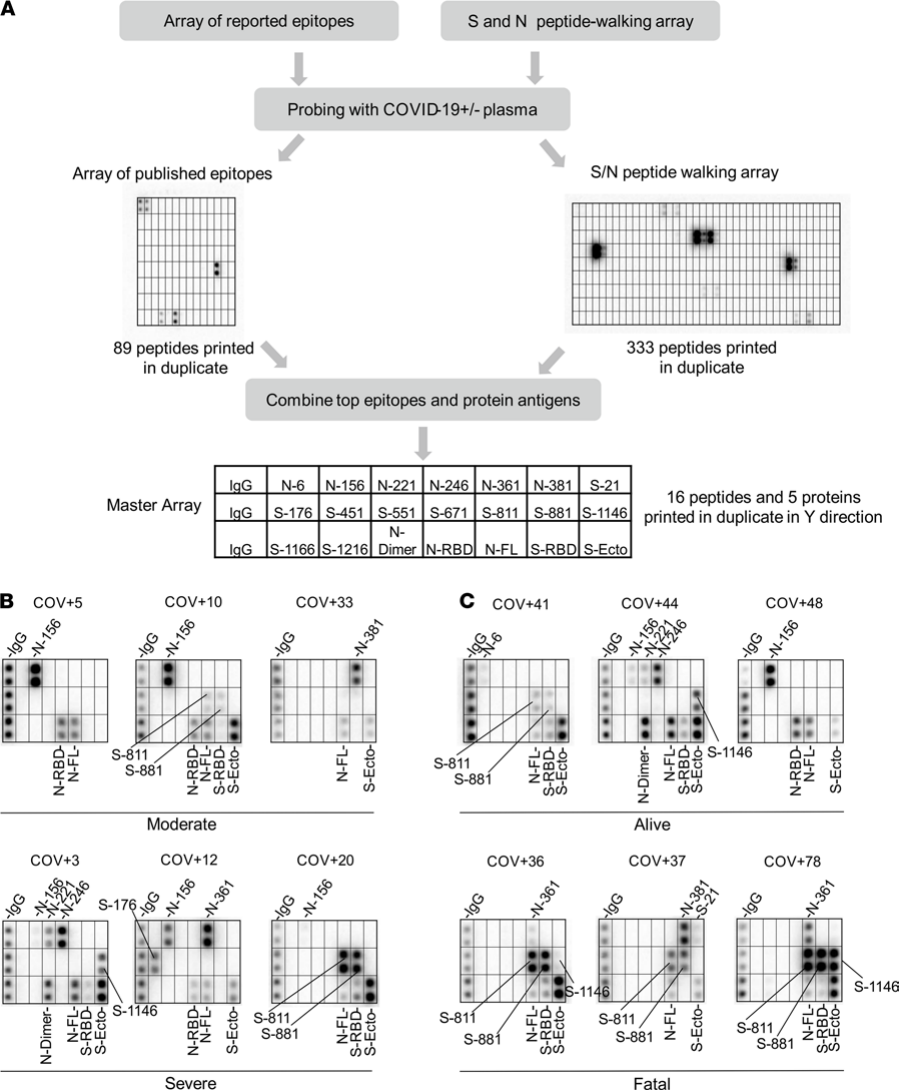
Identification of SARS-CoV-2 epitopes and epitope-resolved antibody profiling. (**A**) Workflow for identifying antigenic epitopes by peptide arrays and the layout of a master array for SARS-CoV-2 antibody profiling. (**B** and **C**) Representative images of epitope-resolved antibody profiles for the different groups of COVID-19 patients (*n* = 65 unique patient samples).

Antibody specificity is determined by epitopes on the protein antigen, including both linear and conformational epitopes (23). Because linear epitopes are small peptides (5–20 residues), they may be identified by screening peptides generated by chemical or genetic means (19, 20, 22). To identify the linear epitopes mediating the SARS-CoV-2 antibody responses, we synthesized peptides representing the candidate epitopes reported in the literature (up to October 2020; refs. 24, 25) and printed the peptides on a nitrocellulose-coated glass slide. The resulting peptide array, containing 89 reported epitopes for the S, N, and membrane proteins (Figure 2A), was probed with patient plasma samples. Intriguingly, we were only able to detect less than 50% of the reported epitopes in our peptide array screens (Supplemental Figure 3 and Supplemental Table 3). While the large discrepancy might be attributed, in part, to the different techniques used for assaying the epitope-antibody interaction, it prompted us to redefine the epitopes using the peptide array approach. To this end, we created a peptide microarray to represent the complete S and N protein sequences. The resulting “peptide-walking” array contained 333 tiled 15-mer peptides with 5-residue overlap between 2 consecutive peptides (Figure 2A).

We screened the peptide microarray with 15 patient plasma samples, including 14 COVID-19 patient samples and 1 SARS-CoV-2^–^ control (Supplemental Figure 4). This led to the identification of 54 potential epitopes from the S and N proteins (Tables 1 and 2). While many of the identified epitopes are likely minor ones based on the corresponding weak IgG-binding signals, some produced strong signals (Supplemental Figure 4 and Tables 1 and 2), suggesting that they might be major epitopes mediating the S or N antibody response. To profile for antibody responses in a systematic manner, we generated a “master array” containing 16 major epitopes selected based on the corresponding IgG signal strength from the peptide-walking array screen. The master array also contained the S and N protein antigens as controls (Figure 2A, Tables 1 and 2, and Supplemental Figure 5).

### Epitope-resolved antibody profiling distinguishes COVID-19 cases based on severity or outcome.

**Figure 3:**
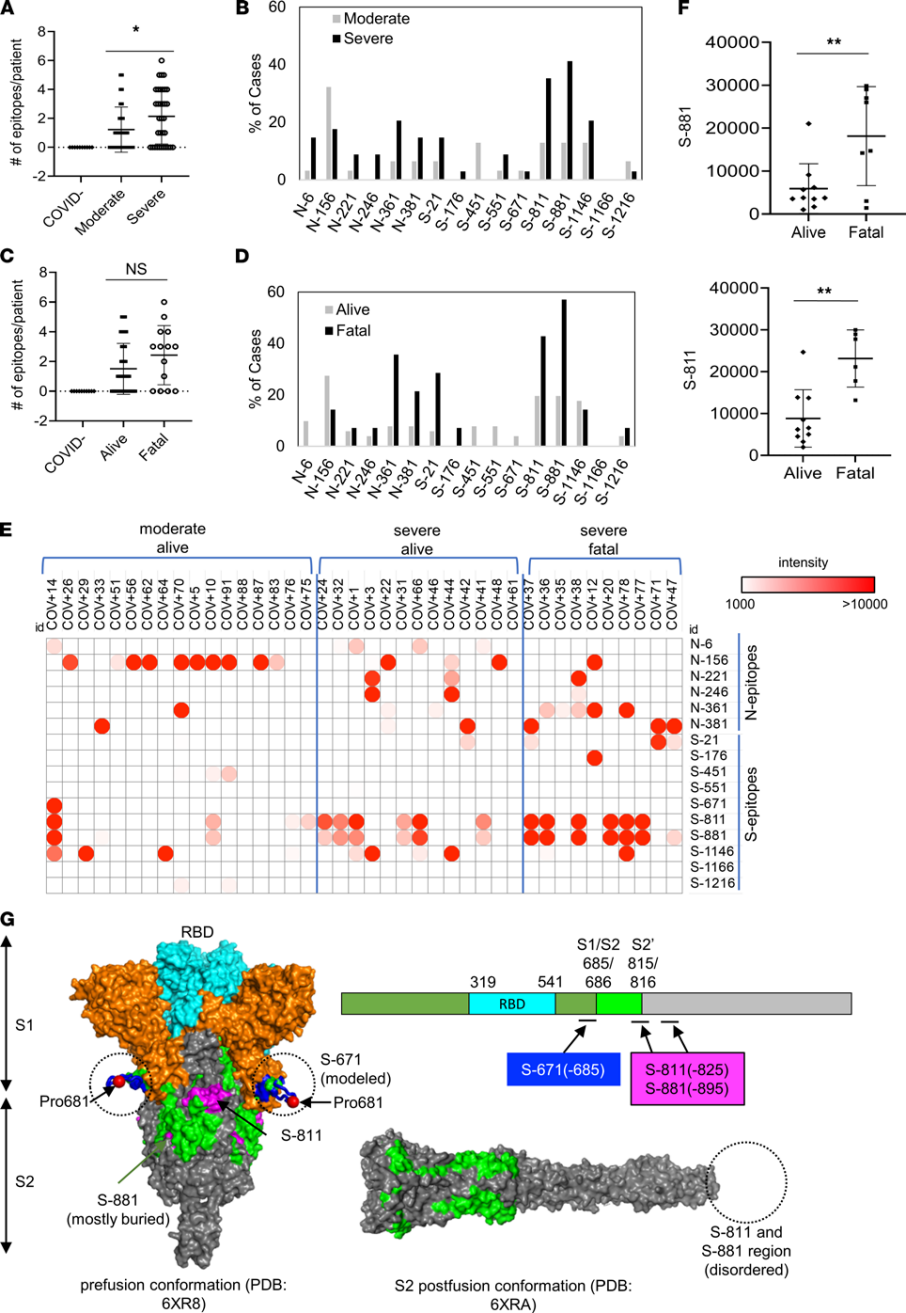
Epitope-specific antibody responses distinguish COVID-19 patients with disparate disease severity and outcome. (**A**) Antibodies from patients with severe disease (*n* = 34) recognized significantly more epitopes than those with moderate conditions (*n* = 31). (**B**) Distribution of epitopes in moderate versus severe cases. (**C**) Number of epitopes/patient in the alive (*n* = 51) versus fatal (*n* = 14) groups. (**D**) Distribution of epitopes in alive versus fatal cases. (**E**) Heatmap representation of epitope-specific antibodies detected by the master array. Note that the heatmap was based on signals detected at low exposure. (**F**) Fatal cases showed significantly stronger antibody responses for the S-811 (alive *n* = 10, fatal *n* = 6) and S-881 (alive *n* = 10, fatal *n* = 8) epitopes. *, *P* < 0.05; **, *P* < 0.002; NS, not significant; unpaired Student’s *t* test with Welch’s correction. (**G**) Structure models to show location of the critical epitopes on the S protein. The epitopes S-671, S-811, and S-881 are shown on the domain structure diagram of S as well as its prefusion (left) and postfusion (right) conformation. The S protein has 2 cleavage sites, S1/S2 and S2′. The S-671 epitope is located at the C-terminus of S1 and disordered in the prefusion cryo–electron microscopy structure (left panel: Protein Data Bank 6XR8). A homology model from the SWISS-MODEL repository was employed to draw an S-671 epitope model in the left panel (colored blue), without cleavage at S1/S2. The Pro681 site is shown with a red sphere. The S2′ cleavage site is located on the S-811 epitope. The S-881 epitope is buried and inaccessible in the prefusion state but is disordered in the postfusion conformation (right panel: Protein Data Bank 6XRA). The S1 region is colored orange, except for the RBD, which is in cyan. The region between the S1/S2 and S2′ cleavage sites is shown in green. The S-811 and S-881 epitopes are colored magenta in the prefusion conformation.

Using the master array, we screened plasma samples from the 89 COVID-19 patients and 9 SARS-CoV-2^–^ control subjects (Figure 2, B and C, and Supplemental Figure 6). Samples with no detectable antibody response (24/89) were subsequently removed from the cohort, resulting in a final cohort of 65 unique patient samples. We found that the plasma from the ICU (severe) group recognized significantly more epitopes than the non-ICU (moderate) group (Figure 3A). Certain epitopes, including S-811, S-881, N-6, and N-361, were detected more frequently in the severe than the moderate cases whereas other epitopes, including S-451 and N-156, showed the opposite trend (Figure 3B). By comparison, the number of IgG-binding epitopes was not significantly different between patients who survived or succumbed to the infection even though the latter group, in general, tended to have antibodies reactive to more epitopes (Figure 3C). Nevertheless, antibodies specific for the S-811, S-881, and N-361 epitopes were found enriched in the fatality group whereas antibodies against N-6, S-451, S-551, and S-671 were detected only in the survivor group (Figure 3D).

In addition to epitope frequency, the intensity of IgG-binding signals to certain epitopes correlated positively or negatively with clinical severity or outcome. In general, we found that moderate cases tended to have stronger antibody responses to N-156 whereas more robust antibody responses against the S-811 and S-881 epitopes were observed for the severe cases (Figure 3E). Indeed, COV+14 was the only case in the moderate group with strong S-811 and S-881 antibodies, which, intriguingly, also featured a robust S-671 antibody response. Overall, the patients with fatal disease were characterized with significantly stronger S-811– or S-881–specific antibodies than those who survived the infection (Figure 3F). This indicates that antibody responses to these epitopes are detrimental to COVID-19 disease progression. The S-811 and S-881 epitopes are located in a region of the S protein buried in the prefusion conformation, which, nevertheless, becomes disordered and exposed following virus fusion with the host cell membrane (Figure 3G). Therefore, it is likely that the production of antibodies specific for the S-811 or S-881 epitopes coincides with the state of the coronavirus undergoing active host cell infection. In contrast, the S-671 epitope, mutated in the UK variant B.1.1.7 (26), is located at the S1/S2 cleavage site critical for virus infection (ref. 27 and Figure 3G).

### Mutations found in SARS-CoV-2 variants alter epitope specificity.

**Figure 4:**
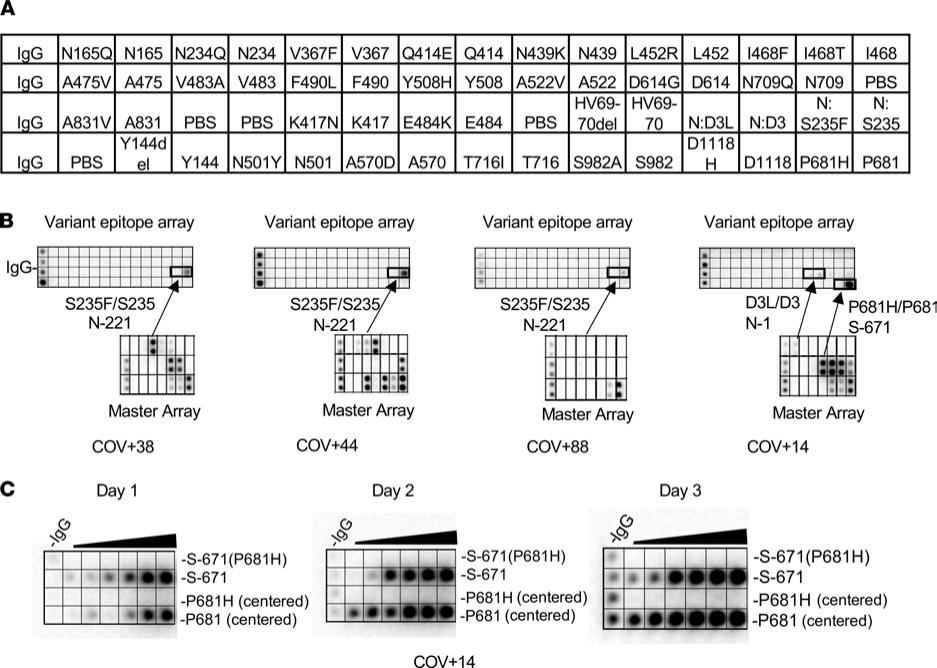
SARS-CoV-2 variants feature mutated epitopes not recognized by antibodies for the corresponding WT epitopes. (**A**) Layout of a SARS-CoV-2 variant epitope array. (**B**) Examples of COVID-19 cases that showed distinct IgG responses to the mutated and WT epitopes (boxed). (**C**) Dilution series of P681/P681H-containing epitopes demonstrating the loss of binding for the mutant epitopes by the patient plasma.

Numerous mutations have been identified in SARS-CoV-2 VOCs, the vast majority of which occur on the S protein (28), which plays a critical role in host cell infection and immune response. The recent emergence of the variants B.1.1.7, P.1, and B.1.351, which have been shown to be more contagious than the original strain, has raised concerns over the efficacy of mRNA vaccines that are used to produce the WT S protein in the recipient (29). We investigated this possibility using peptides representing 28 major S or N missense mutations or deletions identified to date, including those found in the UK variant B.1.1.7 and the South African variant B.1.351, and mutations shown to alter antibody binding in a previous study (ref. 30 and Table 3). A peptide array containing the mutated epitopes and the matching WT epitopes were probed with plasma samples collected prior to October 2020 from patients presumably infected with the original strain of SARS-CoV-2 (Figure 4). Because only a few mutations resided within the identified epitopes (Table 3), the mutated epitope screen was focused on plasma samples that showed robust antibody responses to the corresponding WT epitopes on the master array (Figure 2B). Intriguingly, we found that the mutations either reduced or completely abolished IgG binding for the corresponding epitopes. Of note, substitution of the S235 residue with a Phe in the N-221 epitope, a mutation found in the B.1.1.7 variant, eliminated IgG binding. Similarly, S-671 was identified as a major epitope in the COV+14 patient by the master array. The introduction of the P681H mutation, which has been found in multiple VOCs (31–33), into the S-671 peptide, completely abolished antibody binding. To confirm this finding, we synthesized another version of the S-671 epitope in which the P681 residue and the P681H mutation were placed in the center of the corresponding peptides and printed both versions of the original and mutant peptides in incremental concentrations in an array. This peptide gradient array was then probed with the COV+14 plasma collected on days 1, 2, and 3 of hospitalization. While the original epitopes exhibited increased IgG binding with time, the P681H-mutant epitope did not show detectable antibody binding signal for the same plasma samples. These data indicate that the P681H mutation altered the specificity of the corresponding epitope (S-671) and rendered it unrecognizable by antibodies against the original coronavirus (as the plasma sample was collected prior to the emergence of the B.1.1.7 variant, although the genotype of the virus was not determined).

### A rapid agglutination assay to gauge epitope-specific antibody response.

**Figure 5:**
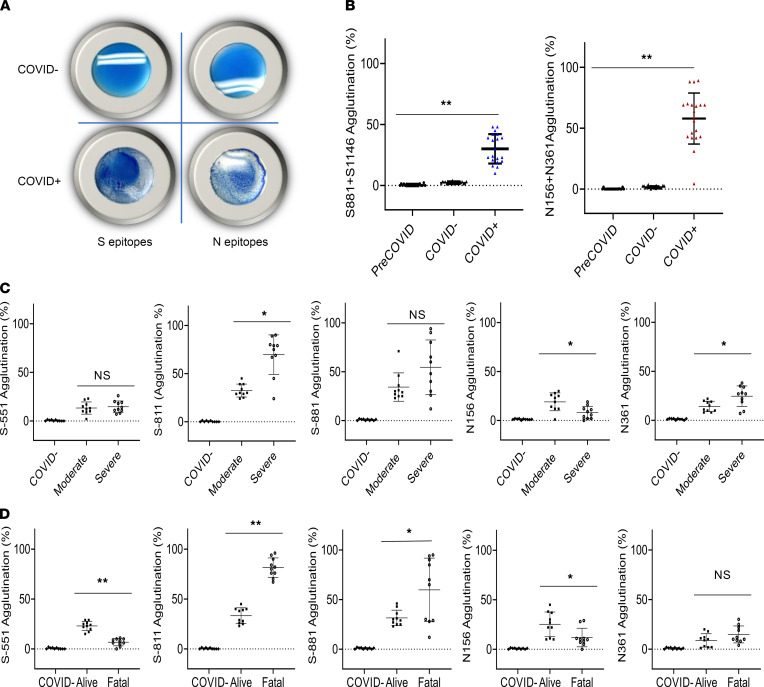
Rapid epitope-dependent agglutination assay for SARS-CoV-2 antibodies effectively differentiates patient groups. (**A**) Latex bead agglutination assay to gauge antibody responses to SARS-CoV-2. The latex beads were coated with 1 or more biotinylated S or N epitope peptides and mixed with SARS-CoV-2–negative (COVID^–^, top) or –positive (COVID^+^, bottom) plasma. The presence of antibodies against the epitopes promoted the agglutination of the latex beads. Images shown were taken after 2 minutes’ incubation at room temperature. (**B**) Epitope-based latex agglutination assay distinguished COVID-19^+^ from COVID-19^–^ or PreCOVID-19 plasma. The epitope peptides used were S-811 and S-1146 from the S and N-156 and N-361 from the N proteins. (**C**) Correlation of disease severity with antibody responses to the S-811, N-156, and N-361 epitopes determined by latex bead agglutination. (**D**) Correlation of disease outcome with antibody responses to the S-551, S-811, S-881, and N-156 epitopes determined by latex bead agglutination. *P* values calculated based on unpaired 1-tailed Student’s *t* test with Welch’s correction (no assumption of equal SD) (*n* = 20 for **B**; *n* = 10 for **C** and **D**). *P < 0.05, **P < 0.002. Error bars represent the SD.

While the epitope peptide array may be used to determine antibody specificity in a systematic manner, it is not suitable for POC testing. Nevertheless, the identification of specific epitopes that are either common to the COVID-19 patients examined or unique to groups with distinct clinical severity or outcome prompted us to develop a rapid test based on these epitopes. Inspired by the principle of antibody-dependent red blood cell agglutination (34), we developed an epitope-dependent agglutination assay to detect epitope-specific antibody response. Specifically, latex beads were coated with streptavidin and conjugated to one or more biotinylated epitope peptides. Antibodies specific to the epitopes were found to induce the agglutination of the corresponding latex beads within minutes (Figure 5A), with the area of agglutination serving as a proxy of antibody titer. In principle, the latex bead agglutination assay is more sensitive than the peptide array because it detects the total antibodies (including IgG, IgM, and IgA) rather than a specific isotype. To develop an epitope test to replace the S and N antigens, we coated the latex beads with the most prominent S or N epitopes. Specifically, latex beads were coated with a mixture of the S-811 and S-1146 (2S) peptides to represent the S antigen or the N-156 and N-361 (2N) peptides to represent the N antigen. When evaluated using plasma samples from individuals who tested positive (COVID^+^) or negative (COVID^–^) for the SARS-CoV-2 virus or samples from healthy donors collected in 2018 (PreCOVID), the 2S- and 2N-based agglutination assays effectively distinguished the COVID^+^ plasma from the COVID^–^ or PreCOVID plasma (Figure 5B).

To determine if the epitope-dependent agglutination assay could differentiate the different patient groups as effectively as the master epitope array, we coated the latex beads with the S epitopes S-811, S-881, or S-551 or the N epitopes N-156 or N-361 and performed agglutination assays on 10 patients/group based on the master array results (i.e., not all patients in the cohort showed antibody responses against a given epitope). While no agglutination was observed for the COVID^–^ plasma, the COVID^+^ plasma promoted the agglutination of the latex beads in an epitope-dependent manner (Figure 5, C and D). We found that the group with severe disease had significantly greater S-811– and N-361–specific antibody responses than that in moderate condition (*P* < 0.05). The reverse was found true for the N-156 epitope (*P* < 0.05). Similarly, significant differences in the antibodies specific for the S-811 (*P* < 0.002), S-881 (*P* < 0.05), S-551 (*P* < 0.002), and N-156 (*P* < 0.05) epitopes were observed between the alive and fatality groups. Notably, a high level of S-811–dependent agglutination was strongly and significantly correlated with patient death whereas even a moderate level of S-551–specific antibody response was correlated significantly with favorable outcome. These data reinforced our findings from the master epitope peptide array screen and identified a group of epitopes, including S-811, S-881, S-551, and N-156, to which antibody responses correlated with clinical severity and outcome of the COVID-19 disease.

### Correlation of epitope-specific antibody response with neutralizing efficiency and disease outcome.

**Figure 6:**
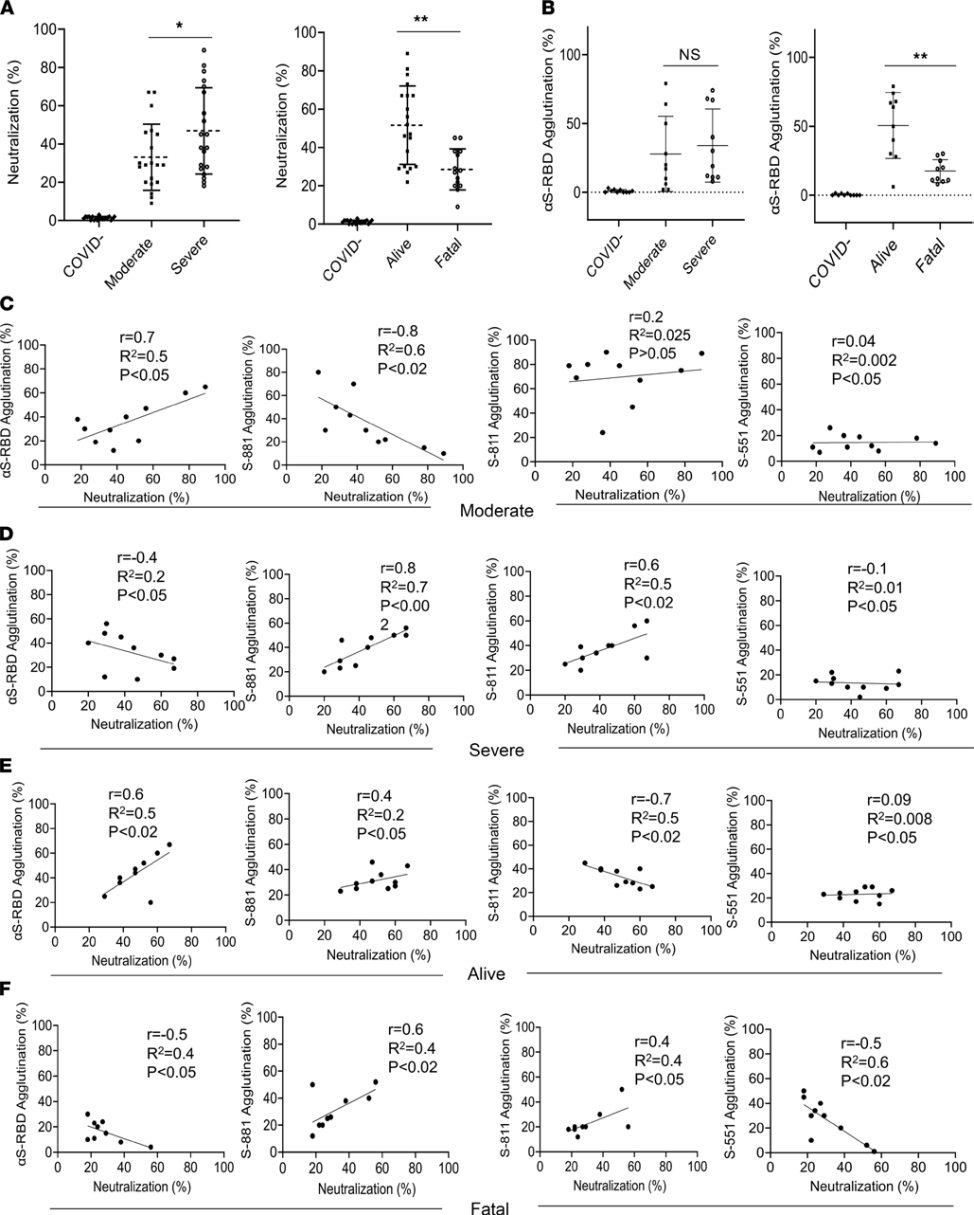
Antibody specificity predicts neutralization efficiency and disease outcome. (**A**) Correlation of neutralization efficiency with clinical severity (left) or outcome (right). *, *P* < 0.05; **, *P* < 0.01. (**B**) Correlation of S-RBD antibody response measured by latex agglutination with COVID-19 severity (left) or outcome (right). **, *P* < 0.01. Error bars represent SD. (**C**–**F**) Pearson’s (*r*) correlation between epitope-dependent agglutination and neutralization. The coefficient of determination (*R*^2^) was calculated based on linear regression analysis. Confidence interval: 95%. The *P* values were calculated using a 2-tailed *t* distribution with *n* – 2 degrees of freedom (*n* = 10). *P* values are shown on each graph.

Because neutralizing antibodies play a pivotal role in the humoral immune response to the SARS-CoV-2 infection, we used a surrogate neutralization assay to measure efficacy of patient plasma in blocking S-RBD binding to its host receptor, angiotensin-converting enzyme 2 (ACE2), in vitro (35). We found that the neutralization efficiency of the plasma in the severe patient group was significantly higher than the group with moderate disease (*P* < 0.05). Intriguingly, the plasma from the fatality group was significantly less efficient in neutralizing S-RBD binding to ACE2 compared with patients who recovered from the infection (*P* < 0.01) (Figure 6A). This suggests that the ability to inhibit the S-RBD-ACE2 interaction, the critical first step in SARS-CoV-2 infection of host cells, dictates disease outcome. Because the identified S epitopes reside outside the RBD domain of the S protein, due perhaps to the possibility that the antibody-RBD recognition involves primarily conformational epitopes (23), we replaced the S epitopes with recombinant RBD and repeated the agglutination assay using the same plasma samples. We found that the S-RBD–dependent antibody response measured by latex agglutination significantly correlated with favorable outcome (*P* < 0.01) (Figure 6B).

Can the S-RBD– or epitope-specific antibody response be used to predict neutralization efficiency? We investigated this possibility by correlating the agglutination data obtained using the S-RBD antigen or the S-881, S-811, or S-551 epitopes with the neutralization data for the same set of patient samples. We found a marked positive correlation between the S-RBD antibody response and neutralization in the moderate or alive group but not in the severe or fatal group (Figure 6, C–F). A negative correlation between the S-811–specific antibody response and neutralization was observed for the moderate group whereas a positive correlation was seen for the severe and fatal groups. Similarly, a positive correlation was observed between the S-811–specific antibody response and neutralization for the severe group. Notwithstanding these observations, we found that the S-551–specific antibody response negatively correlated with neutralization efficiency in the fatal group (Figure 6, C–F). Collectively, these data suggest that a strong S-RBD antibody response together with a weak S-881 or S-811–specific antibody response are correlated with moderate disease and favorable clinical outcome.

## Discussion

The relationship between COVID-19 clinical severity and the humoral immune response is a complex one. It remains poorly understood to date why patients with severe symptoms are characterized with a stronger antibody response, including neutralization antibodies, to SARS-CoV-2 than those who have moderate or mild symptoms (36, 37). This dichotomy suggests that not all antibodies are beneficial. Indeed, while antibodies may mediate the clearance of the virus and virus-infected cells through antibody-dependent cellular cytotoxicity and phagocytosis, they have also been proposed to play a pathogenic role via antibody-dependent enhancement (38). Our epitope-based antibody analysis showed that the antibody responses from different patients are highly varied, and that there is generally no apparent association between the severity of disease presentation and antibody response measured using a protein antigen, including S or N. Therefore, antibody profiling with greater resolution than a simplified S or N antibody classification is needed. Our work, which combines both systematic antibody screen using peptide/protein arrays and rapid antibody assays based on latex particle agglutination, showed that epitope-resolved antibody testing is more sensitive than S/N-based serology tests in discerning antibody specificity and identifying the correlates between humoral immunity and COVID-19 disease severity or outcome.

By identifying and validating the major S and N epitopes to enable epitope-specific antibody testing, our study not only provided support to the notion that linear epitopes play a critical role in mediating antibody responses to SARS-CoV-2 (19, 20, 22, 24, 39), but more importantly, it also demonstrated that the complex antibody responses in individual patients may be deconvoluted by epitope-resolved antibody profiling. Systematic and unbiased antibody profiling using a master array comprising the most prominent epitopes led to several intriguing findings. First, patients with severe disease or poor outcome tend to have antibodies against a large number of epitopes. We showed that these same patients had low levels of neutralizing antibodies. It is therefore possible that the increased production of non-neutralizing antibodies contributed to disease development. Second, all epitopes are not equal, and even the epitopes from the same protein antigen (S or N) may play distinct roles in dictating disease severity and outcome. We have shown not only that S-811 and S-881 are 2 of the most prevalent epitopes but also that a high level of antibodies specific for these epitopes are strongly correlated with severe or fatal diseases. That the S-881– and S-811–specific antibody responses were negatively correlated with neutralization efficiency in patients who had moderate diseases or survived the infection suggests that the corresponding antibodies may promote disease progression by facilitating virus infection of the host cells. Alternatively, antibodies targeting these epitopes may be a surrogate marker for a more robust and potentially excessive immune response causing greater tissue injury. It has been shown that the S-811 epitope is conserved in homologous antigens in several endemic coronaviruses (20). However, our results suggest that the cross-reactive antibodies may not provide protection against SARS-CoV-2. Third, we have shown that mutations found in SARS-CoV-2 variants may directly affect antibody response by altering epitope specificity. This finding demonstrated the flexibility of the epitope peptide array approach to quickly incorporate emerging mutations, thereby providing valuable information of the effect of the mutations and the corresponding VOCs on humoral immunity.

While it has been shown that mutations result in more fit, and likely more contagious, viruses (15, 16, 40), the serological consequences of the mutations found in VOCs are unclear (40). Recent studies have shown reduced binding to therapeutic antibodies or S-specific antibodies for the circulating VOCs B.1.1.7, B.1.351, and P.1 in vitro (41, 42). All 3 VOCs harbor an N501Y mutation within S-RBD, while the B.1.351 and P.1 variants contain 2 additional RBD changes, K417N/T and E484K. These mutations, located at the interfaces of the RBD-ACE2/antibody complexes, have been shown to increase S binding to ACE2 and decrease its recognition by neutralizing antibodies (28, 43–45), leading to the enhanced infection efficacy and transmissibility for the variants. Intriguingly, the same amino acid changes have been shown to alter the corresponding epitopes targeted by neutralizing antibodies (28), thereby providing a potential mechanism of immune escape by reducing or disabling antibody-mediated neutralization (43, 46–48). Besides the RBD, a P681H mutation located in the S1/S2 linker region of the spike has been detected in multiple VOCs (31–33). We found that the P681H mutation in the spike and the S235F in the nucleocapsid rendered the corresponding epitopes completely incapable of binding antibodies generated against the original virus. While it remains to be determined whether these mutations mediate immune escape of the corresponding VOCs in some patients, our findings imply that the P681H mutation may render the WT spike mRNA–based vaccine less effective to those who employ S-671 (which encompasses the mutated residue) as a major epitope. Nevertheless, we note that the P681H and S235F mutations only affected a few individuals in the cohort of patients examined herein while the majority of patients displayed no apparent antibody responses against the corresponding epitopes. This may explain why recent studies have shown the Pfizer and Moderna mRNA vaccines are effective in protecting from infection by the VOCs (49–51). It would be important to investigate in the future, by large-scale epitope-specific antibody profiling, the percentage of the population who employ S-671 as a major epitope. By the same token, prevalence of the N-221 epitope (which contains the S235 residue found mutated in the B.1.1.7 strain) would provide valuable information on the protection of vaccines based on inactivated intact viruses. In the same vein, hundreds of mutations may be examined simultaneously in a peptide array to assess their effect on antibody response, and the epitope array may be readily modified to incorporate emerging mutations. The impact of the mutations on humoral immunity may also involve conformational epitopes that are not recapitulated by the linear epitopes. However, both the master array and the agglutination-based antibody test may be quickly modified to include mutant S proteins. Future studies using a combination of epitope and protein antigen-based assays tailored to the VOCs would provide valuable information on the population penetrance of a given variant and the impact of the associated missense or deletion mutations on antibody-mediated immunity.

While the epitope array may be used to profile antibody response in a systematic manner, the epitope-dependent latex agglutination assay provides a rapid, simple, cost-effective, and accurate serological test that may be suitable for POC antibody testing. The agglutination assay may be carried out with individual epitopes to map the specificity of antibodies or with a mixture of epitopes to test multiple antigens simultaneously. The ease with which to incorporate mutated epitopes or S/N protein antigen in the agglutination assay makes it a nimble yet powerful tool to determine the impact of mutations associated with the VOC on humoral immunity. Although the mRNA-based vaccines have shown superb efficacy, not all vaccine recipients would be protected. It also remains to be determined how long the immunity will last and against which variants. Monitoring vaccinated or recovered individuals over months to years by antibody testing would provide valuable information on the duration of immune responses against SARS-CoV-2, including variants (7). In this regard, the epitope-resolved antibody test may be used to delineate the specific antibodies produced by different individuals, determine persistence of antibody in the circulation over time, assess the efficiency of vaccines, and decipher the effect of the VOCs on the immune system. The agglutination assay, which measures the total antibody response irrespective of the Ig isotypes, provides a unique advantage over serological assays that measure a given isotype because different Ig isotypes have distinct dynamics and evolutionary trajectory over time (18). Longitudinal studies by the epitope-resolved agglutination assay would provide valuable information on the evolution of antibody immunity from vaccination or previous infection.

## Methods

### Patient population and blood sample collection

Spent plasma samples from 89 adult patients (including both male and female) were deidentified prior to transfer from the Core Laboratory (London Health Sciences Center, London, Canada) to a biosafety level 3 (CL3) lab (ImPaKT, Western University) following Transportation of Dangerous Goods guidelines. All plasma samples were heat inactivated at 56°C for 30 minutes at the ImPaKT CL3 facility as per Western University biosafety regulation, then transferred to the testing laboratory. Heat inactivation did not appear to have a significant effect on antibody integrity (52). Heat-inactivated plasma samples were then transferred to the testing laboratory. Clinical data for the whole cohort were not available and therefore not reported in the manuscript. Control samples consisted of patients admitted to hospital due to COVID-19 symptoms but subsequently tested negative by RT-PCR (COVID^–^) as well as samples obtained in 2018, prior to the COVID-19 pandemic.

### Protein microarray

#### Proteins.

The S-ecto (53), expressed in mammalian EXPI293 cells, and N-dimer domain, N-RBD, NSP3-unique, NSP3-ADRP, NSP3-NAB, NSP3-PLPro, NSP4-CTD, NSP5, NSP7, NSP8, NSP9, NSP10, and NSP16 (54) expressed in bacterial *Escherichia coli* cells were supplied by the Toronto Open Access Covid-19 Protein Manufacturing Center (comprising BioZone and the Structural Genomics Consortium, Toronto, Canada) under an Open Science Trust Agreement: http://www.thesgc.org/click-trust The Center received funding from the Toronto COVID-19 Action Fund. See Supplemental Table 1 for a complete list of proteins.

#### Protein array printing.

SARS-CoV-2 proteins were diluted to 0.5–10 μM in PBS with 5% glycerol (IgG control at 200 nM) and aliquots transferred to a 384-well microplate (ArrayIt). A total of 24 copies of the microarray were printed on each nitrocellulose-coated glass slide (ArrayIt) using a VersArray Chipwriter Pro (Bio-Rad) equipped with a Stealth 15XB microarray quill pin (ArrayIt). Spot-to-spot distance was 850 μm with 2 reprints of the same spot and all spots printed in duplicate in the *y* dimension. A dwell time of 0.1 seconds was used for each spot with an approach speed of 12.5 mm/s. Samples were printed at room temperature and subsequently stored at 4°C until time of probing.

### Peptide microarray

#### Peptide synthesis.

Peptides were synthesized on Tentagel resin on an Intavis MultiPep RSi peptide synthesizer using *N*-(9-fluorenyl) methoxycarbonyl chemistry. All peptides were synthesized with biotin at the N-terminus followed by an aminohexanoic acid and Gly-Gly spacer. A walking array of peptides with 15–amino acid length and 5–amino acid overlap spanning the full sequence of SARS-CoV-2 S (UniProt Protein Accession P0DTC2.1) and N proteins (UniProt Protein Accession P0DTC9.1) were synthesized for array printing. Peptides reported in a previous publication (24) as well as epitopes predicted using bioinformatics (25) were synthesized and printed to create the literature-reported peptide array. Peptides encompassing mutation sites reported in SARS-CoV-2 variants were synthesized as described above for the variant peptide array (Table 2).

#### Peptide array printing.

Peptides were printed as neutravidin complexes on nitrocellulose-coated slides (ArrayIt) by mixing 10 μM neutravidin with an excess (by 4-fold) of peptide that was diluted in PBS, and aliquots were transferred to a 384-well microplate (ArrayIt) along with IgG printing control, S-RBD, full-length N, N-RBD, and N-dimer proteins. Two copies of the walking microarray, 3 copies of the literature-reported microarray, or 8 copies of the variant array were printed on each nitrocellulose-coated glass slide using a VersArray Chipwriter Pro (Bio-Rad) equipped with a Stealth 15XB microarray quill pin (ArrayIt). Spot-to-spot distance was 750 μm with 2 reprints of the same spot and all spots printed in duplicate in the *y* dimension. A dwell time of 0.1 seconds was used for each spot with an approach speed of 12.5 mm/s. Samples were printed at room temperature and subsequently stored at 4°C until time of probing.

### Protein and peptide array probing

Microarray slides were briefly rinsed twice with Tris-buffered saline containing Tween 20 (TBST: 0.1 M Tris-HCl at pH 7.4, 150 mM NaCl, and 0.1% Tween 20) to wet the surface and then incubated for 2 hours with ChonBlock ELISA blocking and antibody dilution buffer (Chondrex Inc). Slides were briefly rinsed with TBST, then inserted into an ArraySlide 24-chamber hybridization cassette (The Gel Company) for the proteome array or ProPlate Multi-Well Chamber (Grace Bio-Labs) for the peptide arrays and incubated with plasma from NAT-confirmed SARS-CoV-2–positive and –negative patients (1:250 dilution in ChonBlock). Slides were then rinsed quickly 3 times followed by 3 washes, 5 minutes each, with TBST before probing with goat anti–human IgG HRP antibody at 1:10,000 (MilliporeSigma, AP113P) in ChonBlock for 1 hour. The wash step was repeated as above; then the HRP signal was visualized on a ChemiDoc XRS+ Imager (Bio-Rad) using Clarity ECL Substrate (Bio-Rad). Slides were incubated with ECL solution for 30 seconds; then 15 images were taken incrementally from 1 to 60 seconds. All incubation steps were performed at room temperature using a rocker for agitation of the sample. Antibodies were used in place of plasma to confirm protein printing as follows: anti–S-RBD (Novus Biologicals clone CR3022, NBP2-90980) at 1:1000 followed by goat anti–human IgG HRP (same as above) and anti-nucleocapsid (Thermo Fisher Scientific PA5-81794) at 1:1000 followed by goat anti-rabbit IgG HRP (Bio-Rad, 1721050) at 1:10,000. All antibodies were diluted in ChonBlock.

### Array quantification

Peptide-walking arrays, literature-reported epitope peptide arrays, and the master epitope arrays were quantified using ImageJ software (NIH) (55). Images were first inverted and converted to 8 bit. Background was subtracted using a rolling ball radius of 25 pixels. Intensities were normalized to IgG control and ranked by normalized signal intensity. Peptides with strongest intensity or most frequently observed were selected for creation of the master array. To determine the percentage of cases and number of epitopes per patient, high exposure (60 seconds) images were captured to visualize very-low-intensity spots. Due to oversaturation at this exposure time, lower exposure (5–10 seconds) images were used for quantification purposes. For the master array quantification, signals within 2 SDs of the mean background intensity at lower exposure were omitted from statistical analysis. Patient samples with no detectable antibody response (24 out of 89 patients) were also omitted from statistical analysis.

### Preparation of SARS-CoV-2 peptide antigen-conjugated latex particles and peptide antigen-based agglutination assay

Blue-dyed, carboxylate-modified, streptavidin-polystyrene, latex beads, 0.25 μm in diameter, or blue-dyed, polystyrene, latex beads, 0.8 μm in diameter, were purchased from MilliporeSigma (L6155, L1398). Carboxylate-modified latex-streptavidin or neutravidin-coated polystyrene beads were suspended at 2.5% (w/v) using assay buffer, 0.025 M 2-[*N*-Morpholino] ethanesulfonic acid–Tween 20 buffer (0.05% pH 6.0). Synthetic biotin-labeled SARS-CoV-2 peptides were suspended in the same assay buffer at the concentration of 500 μg/mL. The biotin peptides were incubated with streptavidin-latex beads for 1 hour at room temperature. The epitope peptide-conjugated latex bead complex was washed twice with PBS buffer (135 mM NaCl, 2.6 mM KCl, 8 mM Na_2_HPO_4_, and 1.5 mM KH_2_PO_4_, pH 7.4) by mixing and centrifuging the latex suspension at 5000*g* for 10 minutes at room temperature. The peptide antigen-bead conjugate was blocked for 30 minutes at room temperature in PBS containing 3% bovine serum albumin (BSA). The conjugate was then resuspended at 2.5% (w/v) in PBS containing 1% BSA and stored at 4°C until use. For the agglutination assay, 5 μL plasma was mixed with 25 μL peptide-conjugated latex beads (2.5%, w/v) per assay as described in the full protein antigen agglutination assay section.

### Agglutination assay for SARS-CoV-2 antibody testing and data interpretation

For the agglutination assay, 10 samples were chosen for each epitope comparison based on the presence of antibody responses on the microarray screens. A total of 5 μL plasma was mixed with 25 μL antigen-coated beads (2.5%, w/v) per assay. The agglutination was allowed to proceed for 2 minutes at room temperature before imaging with a camera. The relative degree of agglutination induced by the SARS-CoV-2 antibody was measured by the area of clump formation based on the corresponding image. The image analysis software Qupath (v0.1.2) was used (https://qupath.github.io/), and quantification was done by calculating the percentage of agglutination based on estimated agglutination/clumps area (mm^2^) relative to the total latex reaction area. We used 5% agglutination as the cutoff for antibody positivity.

### S-RBD–ACE2 binding ELISA surrogate neutralization assay

Biotin-ACE2 (1 μg/mL) was added to an S-RBD–coated plate after blocking and incubated for 1 hour at room temperature. The wells were washed 3 times with TBST (20 mM Tris, 150 mM NaCl, 0.1% Tween 20) to remove unbound biotin-ACE2. Streptavidin-HRP (Thermo Fisher Scientific PI21124), with 1000-fold dilution in ChonBlock blocking buffer, was then added to each well and incubated for 1 hour at room temperature. The wells were washed 3 times with TBST; 3,3′,5,5′-tetramethylbenzidine substrate (Thermo Fisher Scientific, N301) was added for reaction development; and 0.18 M H_2_SO_4_ was used to stop the reaction. Absorbance at 450 nm was measured to detect the S-RBD–bound ACE2. To determine the neutralization efficacy of the patient plasma, the plasma was diluted 1:100 and incubated with S-RBD–coated wells (blocked) for 1 hour at room temperature. The wells were washed 3 times with TBST. Biotin-ACE2 was then added to the wells and incubated for 1 hour at room temperature followed by washing, reaction development, and detection as described above.

### Statistics

All statistical analyses were performed using GraphPad Prism 9 software. Significance was determined using unpaired 1-tailed Student’s *t* test with Welch’s correction and 1-way ANOVA with Geisser-Greenhouse correction and *P* ≤ 0.05 was considered significant. Error bars represent the SD.

### Study approval

Blood samples were collected following a protocol (study number: 116284) approved by the Research Ethics Board of Western University.

## Author contributions

SSCL and ICY conceived the study. SSCL, CV, SE, and XL designed the study. CV performed the protein and peptide array screens and data analysis. SE performed the agglutination and neutralization assay and data analysis. XL and SZ synthesized the peptides. SA, PS, AS, AH, FY, TS, EE, PL, PG, and TH contributed to subcloning, expression, and purification of SARS-CoV-2 proteins and human ACE2. MJK, LL, HA, BDH, VB, CMM, MS, and DF contributed to patient recruitment, blood sample collection, and NAT and ELISA testing. TK and BCY contributed to data analysis and interpretation. SSCL, CV, and SE wrote the manuscript with input from BCY, ICY, SA, MJK, and DDF.

## Supplementary Material

Supplemental data

## Figures and Tables

**Table 1:**
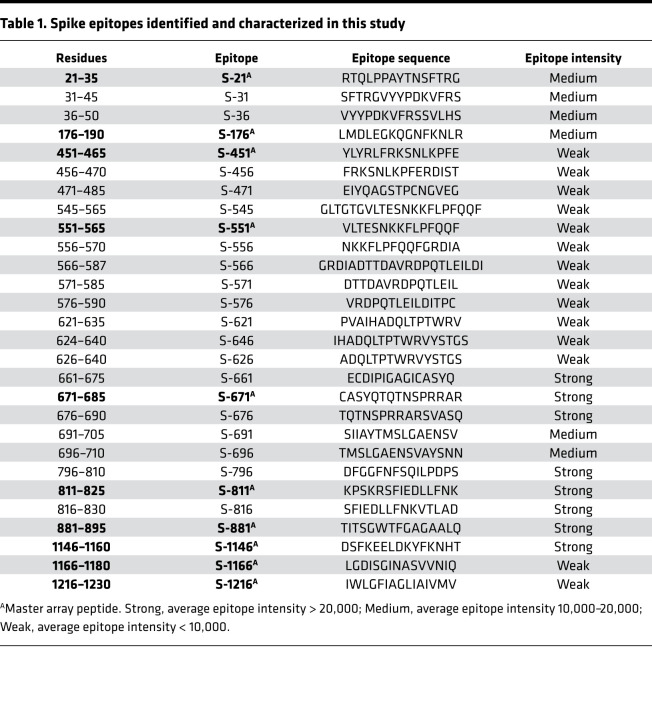
Spike epitopes identified and characterized in this study

**Table 2:**
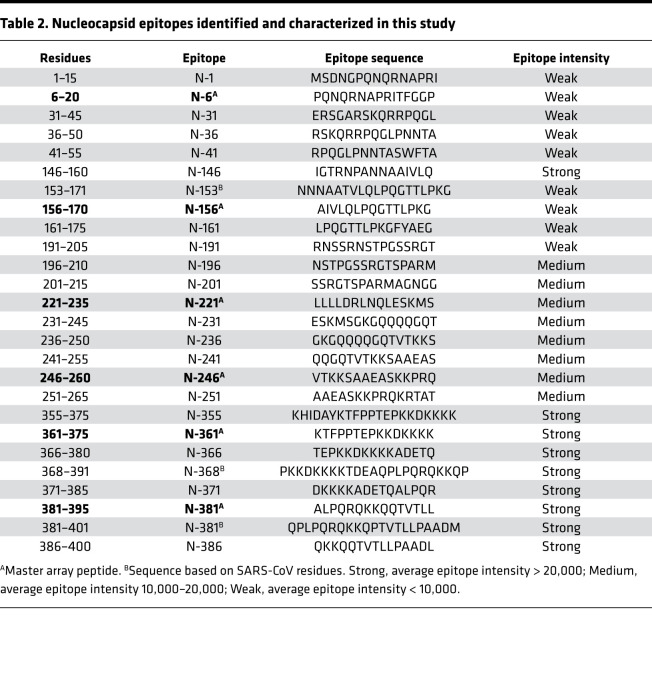
Nucleocapsid epitopes identified and characterized in this study

**Table 3:**
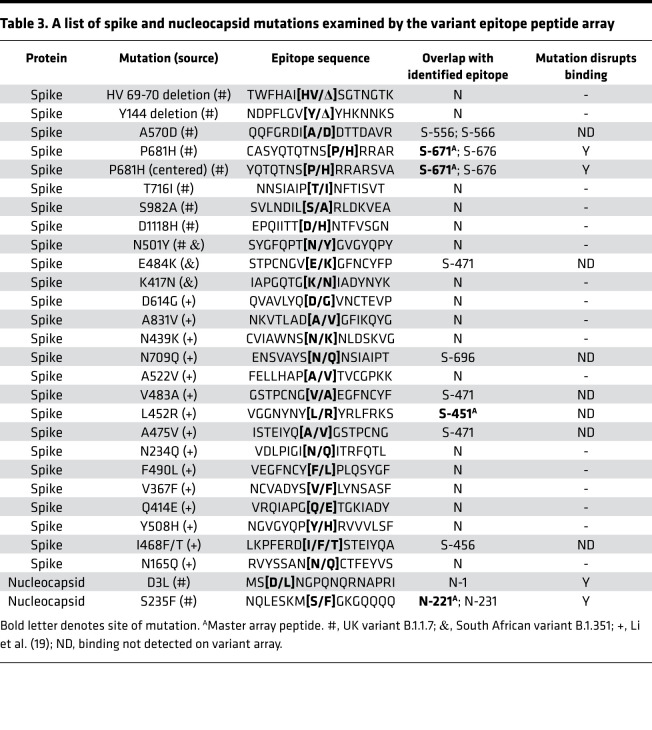
A list of spike and nucleocapsid mutations examined by the variant epitope peptide array

## Version 1 06/03/2021

In-Press Preview

## Version 2 07/08/2021

Electronic publication
