# Supplementary material for: Epitope-specific antibody responses differentiate COVID-19 outcomes and variants of concern
Source: JCI Insight. 2021 Jul 8;6(13):e148855. doi: 10.1172/jci.insight.148855 (PMC8410046; doi:10.1172/jci.insight.148855)
Supplement: Supplemental data [file jciinsight-6-148855-s034.pdf]

Epitope-specific antibody responses differentiate COVID-19 outcome and variants of concern

Courtney Voss[1,9], Sally Esmail[1,9], Xuguang Liu[1], Michael J. Knauer[2], Suzanne Ackloo[3], Tomonori Kaneko[1], Lori Lowes[2], Peter Stogios[4], Almagul Seitova[3], Ashley Hutchinson[3], Farhad Yusifov[3], Tatiana Skarina[4], Elena Evdokimova[4], Peter Loppnau[4], Pegah Ghiabi[4], Taraneh Haijan[4], Shanshan Zhong[1], Husam Abdoh[2], Benjamin D. Hedley[2], Vipin Bhayana[2], Claudio M. Martin[5,6], Marat Slessarev[5,6], Benjamin Chin-Yee[7], Douglas D. Fraser[5,6,8], Ian Chin-Yee[2] and Shawn S-C. Li[1*]

[1]Department of Biochemistry and [2]Department of Pathology and Laboratory Medicine, Western University, London, Ontario N6A 5C1; [3]Structural Genomics Consortium and [4]Department of Chemical Engineering and Applied Chemistry, University of Toronto, 101 College St., Toronto, Ontario M5G 1L7; [5]Department of Medicine, Western University, London, Ontario N6A 5C1; [6]London Health Sciences Centre, 800 Commissioners Rd E, London, Ontario N6A 5W9; [7]Divison of Hematology and [8]Department of Pediatrics, Western University, London, Ontario N6A 5C1 Canada.

[9]These authors contributed equally to this work.

*Corresponding author: Email: sli@uwo.ca

Supplemental Table 1. Recombinant proteins and printing concentrations on the proteome array

Protein	Residues	Printed Concentration
IgG	Equitech Bio Inc<br>SLH66-0001	200nM
Spike Receptor Binding Domain	ThermoFisher Scientific RP-87678<br>319-541	14μM
Spike Ectodomain	1-1208	0.5μM
NSP3-unique	Replicase polyprotein 1ab 413-676	10μM
NSP3-ADRP	Replicase polyprotein 1ab 1024-1192	10μM
NSP3-PLPro	Replicase polyprotein 1ab 1561-1878	10μM
NSP3-nucleic acid binding domain	Replicase polyprotein 1ab 1867-2034	10μM
NSP4-C terminal Domain	Replicase polyprotein 1ab 3173-3569	10μM
NSP5	Replicase polyprotein 1ab 3264-3569	10μM
NSP7	Replicase polyprotein 1ab 3860-3942	10μM
NSP8	Replicase polyprotein 1ab 3943-4140	10μM
NSP9	Replicase polyprotein 1ab 4141-4253	10μM
NSP10	Replicase polyprotein 1ab 4254-4392	10μM
NSP16	Replicase polyprotein 1ab 6799-7096	10μM
Nucleocapsid<br>(full length)	RayBiotech 230-01104<br>1-419	5μM
Nucleocapsid Dimerization Domain	247-364	5μM
Nucleocapsid RNA Binding Domain	47-173	5μM

Supplemental Table 2. Patient cohort used in this study

Sample ID	ICU	Outcome	Severity
COV+5	N	Alive	Moderate
COV+7	N	Alive	Moderate
COV+8	N	Alive	Moderate
COV+10	N	Alive	Moderate
COV+11*	N	Alive	Moderate
COV+14	N	Alive	Moderate
COV+17*	N	Alive	Moderate
COV+18*	N	Fatal	Moderate
COV+19*	N	Alive	Moderate
COV+26	N	Alive	Moderate
COV+27	N	Alive	Moderate
COV+28	N	Alive	Moderate
COV+29	N	Alive	Moderate
COV+30*	N	Alive	Moderate
COV+33	N	Alive	Moderate
COV+34	N	Alive	Moderate
COV+51	N	Alive	Moderate
COV+52	N	Alive	Moderate
COV+53*	N	Alive	Moderate
COV+55	N	Alive	Moderate
COV+56	N	Alive	Moderate
COV+57*	N	Alive	Moderate
COV+58	N	Alive	Moderate
COV+59	N	Alive	Moderate
COV+62	N	Alive	Moderate
COV+63	N	Alive	Moderate
COV+64	N	Alive	Moderate
COV+65*	N	Alive	Moderate
COV+67	N	Alive	Moderate
COV+70	N	Alive	Moderate
COV+72	N	Alive	Moderate
COV+73	N	Alive	Moderate
COV+74	N	Alive	Moderate
COV+75	N	Alive	Moderate
COV+76	N	Alive	Moderate
COV+81*	N	Alive	Moderate
COV+82*	N	Alive	Moderate
COV+83	N	Alive	Moderate
COV+84*	N	Alive	Moderate
COV+85*	N	Alive	Moderate
COV+86*	N	Alive	Moderate

COV+87	N	Alive	Moderate
COV+88	N	Alive	Moderate
COV+89*	N	Alive	Moderate
COV+90*	N	Alive	Moderate
COV+91	N	Alive	Moderate
COV+1	Y	Alive	Severe
COV+2	Y	Fatal	Severe
COV+3	Y	Alive	Severe
COV+4	Y	Alive	Severe
COV+6	Y	Alive	Severe
COV+9	Y	Fatal	Severe
COV+12	N	Fatal	Severe
COV+13*	N	Fatal	Severe
COV+15*	Y	Fatal	Severe
COV+16*	N	Fatal	Severe
COV+20	Y	Fatal	Severe
COV+22	Y	Alive	Severe
COV+24	Y	Alive	Severe
COV+25*	Y	Alive	Severe
COV+31	Y	Alive	Severe
COV+32	Y	Alive	Severe
COV+35	Y	Fatal	Severe
COV+36	Y	Fatal	Severe
COV+37	Y	Fatal	Severe
COV+38	Y	Fatal	Severe
COV+39	Y	Alive	Severe
COV+40	Y	Fatal	Severe
COV+41	Y	Alive	Severe
COV+42	Y	Alive	Severe
COV+43	Y	Alive	Severe
COV+44	Y	Alive	Severe
COV+45	Y	Alive	Severe
COV+46	Y	Alive	Severe
COV+47	Y	Fatal	Severe
COV+48	Y	Alive	Severe
COV+49	Y	Fatal	Severe
COV+50	Y	Fatal	Severe
COV+54	Y	Alive	Severe
COV+60	Y	Alive	Severe
COV+61	Y	Alive	Severe
COV+66	Y	Alive	Severe
COV+68*	Y	Alive	Severe
COV+69*	N	Fatal	Severe

COV+71	Y	Fatal	Severe
COV+77	Y	Fatal	Severe
COV+78	N	Fatal	Severe
COV+79*	Y	Fatal	Severe
COV+80*	N	Fatal	Severe
COV-1	N	Alive	Negative
COV-2	N	Alive	Negative
COV-3	N	Alive	Negative
COV-4	N	Alive	Negative
COV-5	N	Alive	Negative
COV-6	N	Alive	Negative
COV-7	N	Alive	Negative
COV-8	N	Alive	Negative
COV-9	N	Alive	Negative
COV-10	N	Alive	Negative
COV-11	N	Alive	Negative
COV-35	N	Alive	Negative
COV-36	N	Alive	Negative
COV-37	N	Alive	Negative
COV-38	N	Alive	Negative
COV-39	N	Alive	Negative
COV-40	N	Alive	Negative
COV-41	N	Alive	Negative
COV-42	N	Alive	Negative
COV-43	N	Alive	Negative
COV-44	N	Alive	Negative
COV-45	N	Alive	Negative
COV-46	N	Alive	Negative
COV-47	N	Alive	Negative
COV-48	N	Alive	Negative
COV-49	N	Alive	Negative
COV-50	N	Alive	Negative

*No antibodies detected; sample was removed from analysis. Moderate cases defined as those with intensive care unit (ICU) admission; Severe cases defined as patients admitted to ICU or fatal; cases defined as Negative were determined by quantitative PCR analysis for SARS-CoV-2.

Supplemental Table 3. A list of literature reported epitopes examined in this study

Protein	Residues	Sequence	Source	Confirmed on Peptide Array
Spike	21-35	RTQLPPAYTNSFTRG	Δ	Y
Spike	181-195	GKQGNFKNLREFVFK	Δ	N
Spike	351-365	YAWNRKRISNCVADY	Δ	N
Spike	451-465	YLYRLFRKSNLKPFE	Δ	Y
Spike	801-815	NFSQILPDPSKPSKR	Δ	N
Spike	811-825	KPSKRSFIEDLLFNK	Δ	Y
Spike	881-895	TITSGWTFGAGAALQ	Δ	Y
Spike	1041-1055	DFCGKGYHLMSFPQS	Δ	N
Spike	1091-1105	REGVFVSNGTHWFVT	Δ	N
Spike	612-623	YQDVNCTEVPVA	δ	N
Spike	491-504***	PLQSYGFQPTNGVGT	δ	N
Spike	802-819	FSQILPDPSKPSKRSFIE	δ	Y
Spike	888-902	FGAGAALQIPFAMQM	δ	N
Spike	895-909	QIPFAMQMAYRFNGI	δ	N
Spike	287-305	DAVDCALDPLSETKCTLKS	δ	N
Spike	306-318***	FTVEKGIYQTSNGGGTPCS	δ	N
Spike	588-598***	NGGGTPCSFGGVSVI	δ	N
Spike	524-545	VCGPKKSTNLVKNKCVNFNFN	δ	N
Spike	545-565	GLTGTGVLTESNKKFLPFQQF	δ	Y
Spike	566-587	GRDIADTTDAVRDPQTLEILDI	δ	Y
Spike	601-611	GTNTSNQVAVL	δ	N
Spike	624-640	IHADQLTPTWRVYSTGS	δ	Y
Nucleocapsid	61-75	KEDLKFPRGQGVPIN	Δ	N
Nucleocapsid	91-105	TRRIRGGDGKMKDLS	Δ	N
Nucleocapsid	161-175	LPQGTTLPKGFYAEG	Δ	Y
Nucleocapsid	201-215	SSRGTSPARMAGNGG	Δ	Y
Nucleocapsid	221-235	LLLLDRLNQLESKMS	Δ	Y
Nucleocapsid	251-265	AAEASKKPRQKRTAT	Δ	Y
Nucleocapsid	311-325	ASAFFGMSRIGMEVT	Δ	N
Nucleocapsid	361-375	KTFPPTEPKKDKKKK	Δ	Y
Nucleocapsid	371-385	DKKKKADETQALPQR	Δ	Y
Nucleocapsid	381-395	ALPQRQKKQQTVTLL	Δ	Y
Nucleocapsid	391-405	TVTLLPAADLDDFSK	Δ	N
Nucleocapsid	41-61	RPQGLPNNTASWFTALTQHGK	δ	Y
Nucleocapsid	153**	NNNAATVLQLPQGTTLPKG	δ	Y
Nucleocapsid	355-375	KHIDAYKTFPPTEPKKDKKKK	δ	Y
Nucleocapsid	381-401**	QPLPQRQKKQPTVTLLPAADM	δ	Y
Nucleocapsid	368-391**	PKKDKKKKTDEAQPLPQRQKKQP	δ	Y

Δ Wang *et al*. (2020) *ACS Cent Sci* **6**: 2238-2249; δ Grifoni *et al*. (2020) *Cell Host Microbe* **27**: 671-680 e2. ** Based off SARS CoV sequence; *** Non-linear peptide from Grifoni et al. 2020

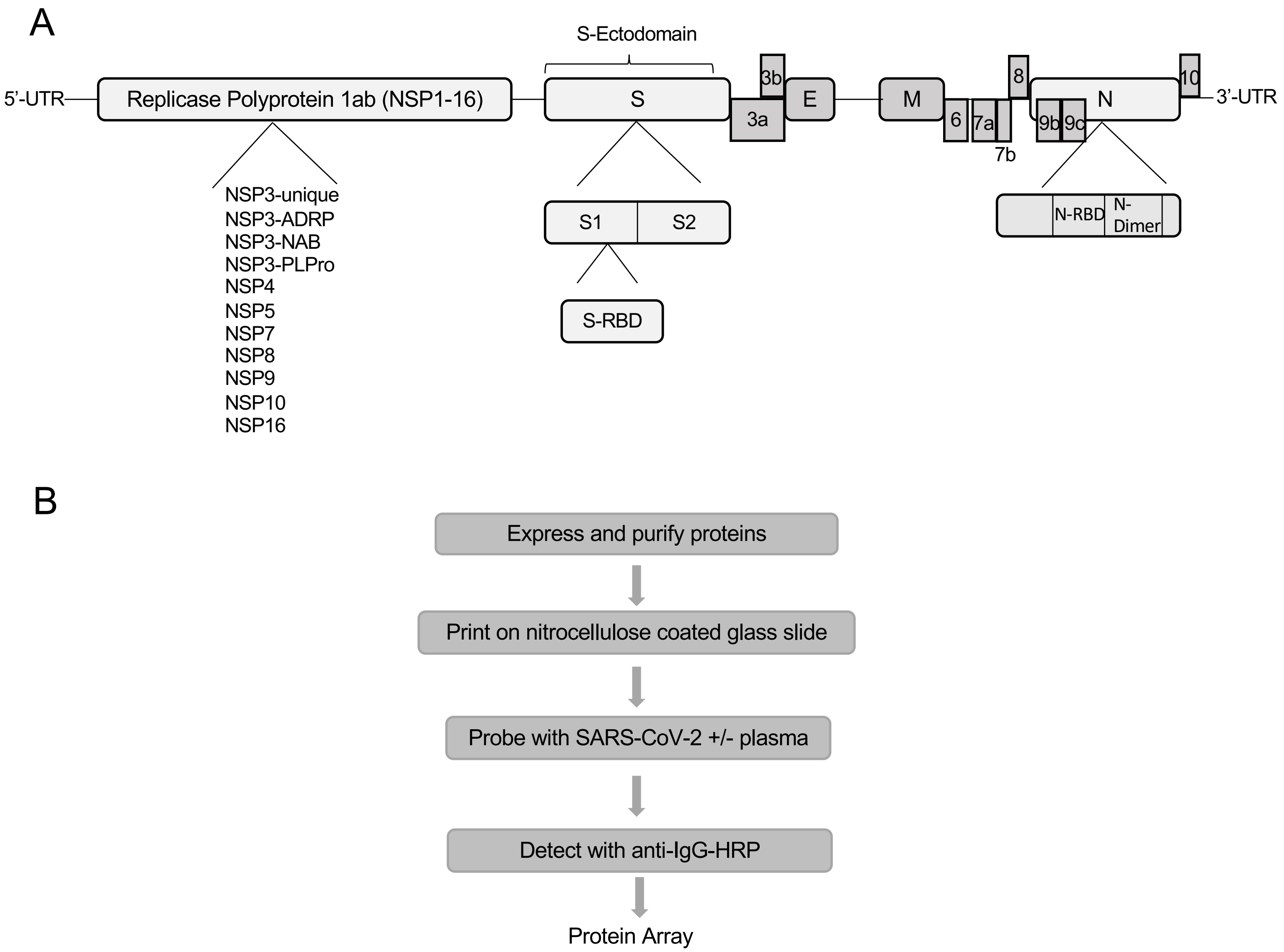

C

IgG	PBS	S-RBD	PBS	S-Ecto	PBS	NSP3 unique	NSP3 ADRP
IgG	NSP3 PLPro	NSP3 NAB	NSP4	NSP5	NSP7	NSP8	NSP9
IgG	NSP10	NSP16	PBS	N-FL	PBS	N-Dimer	N-RBD

16 proteins printed in duplicate in Y direction

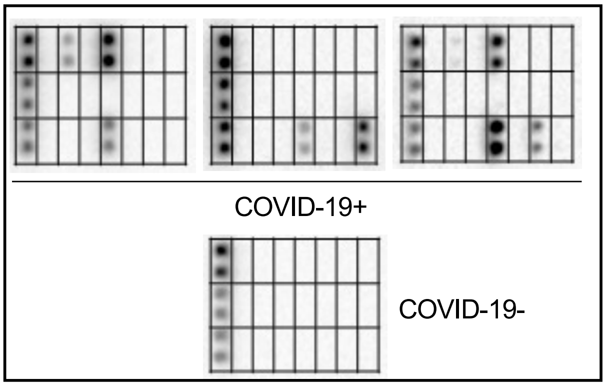

Supplemental Figure 1. Characterizing SARS-CoV-2 antibody responses using a proteome array.
(A) A schematic diagram showing the major proteins of SARS-CoV-2. (B) Workflow for the production and screening of a SARS-CoV-2 proteome array. (C) Left, layout of the SARS-CoV-2 proteome array; Right, representative images of the proteome array probed with SARS-CoV-2-positive or -negative (based on NAT) plasma.

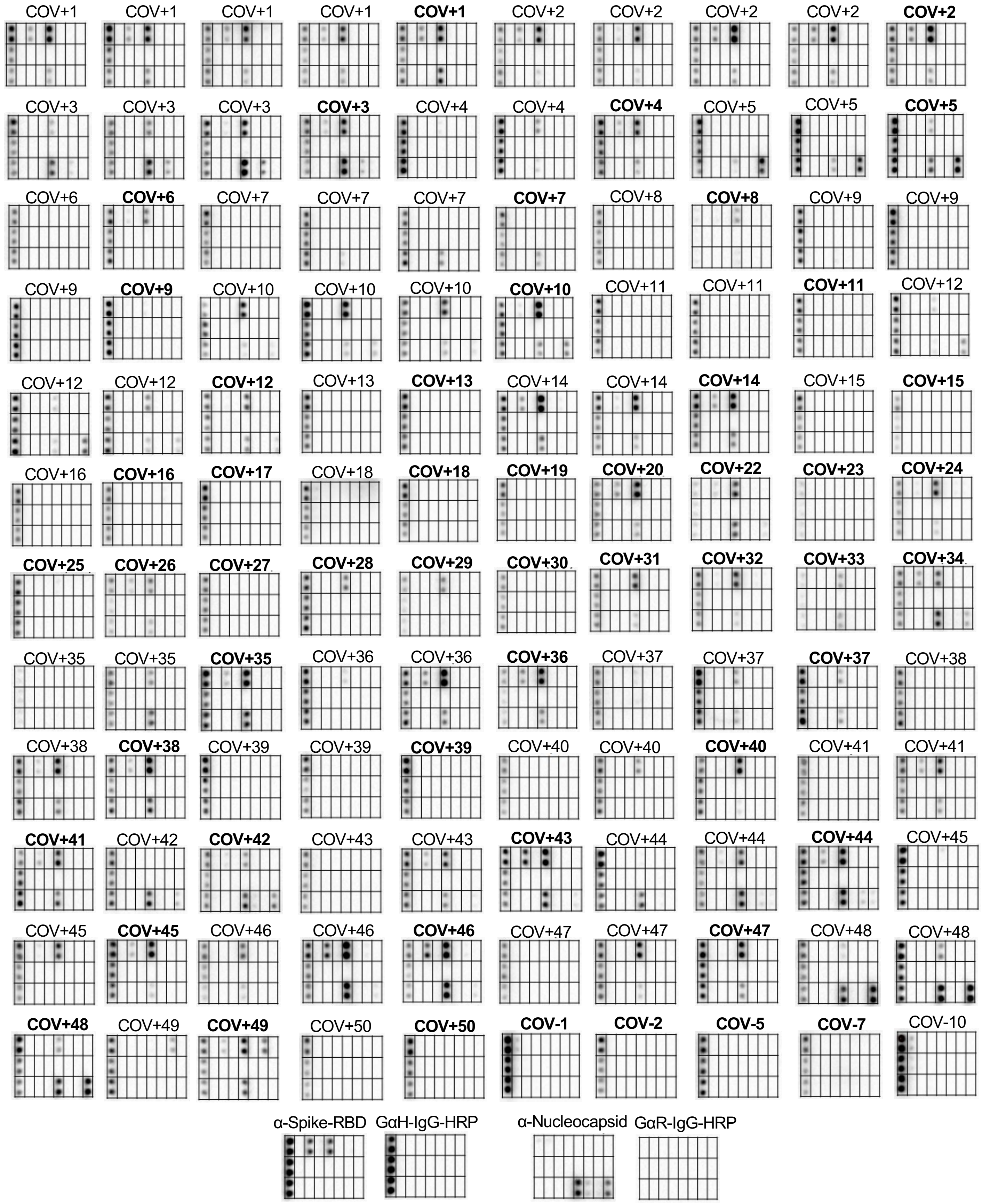

Supplemental Figure 2. Antibody responses profiled using the SARS-CoV-2 proteome array. Twenty-four copies of the grid were printed on a single nitrocellulose coated glass slide. Serial patient samples are shown sequentially and bold are the patient samples subsequently analyzed on the master array. All images shown were taken with 5.2 second exposure time.

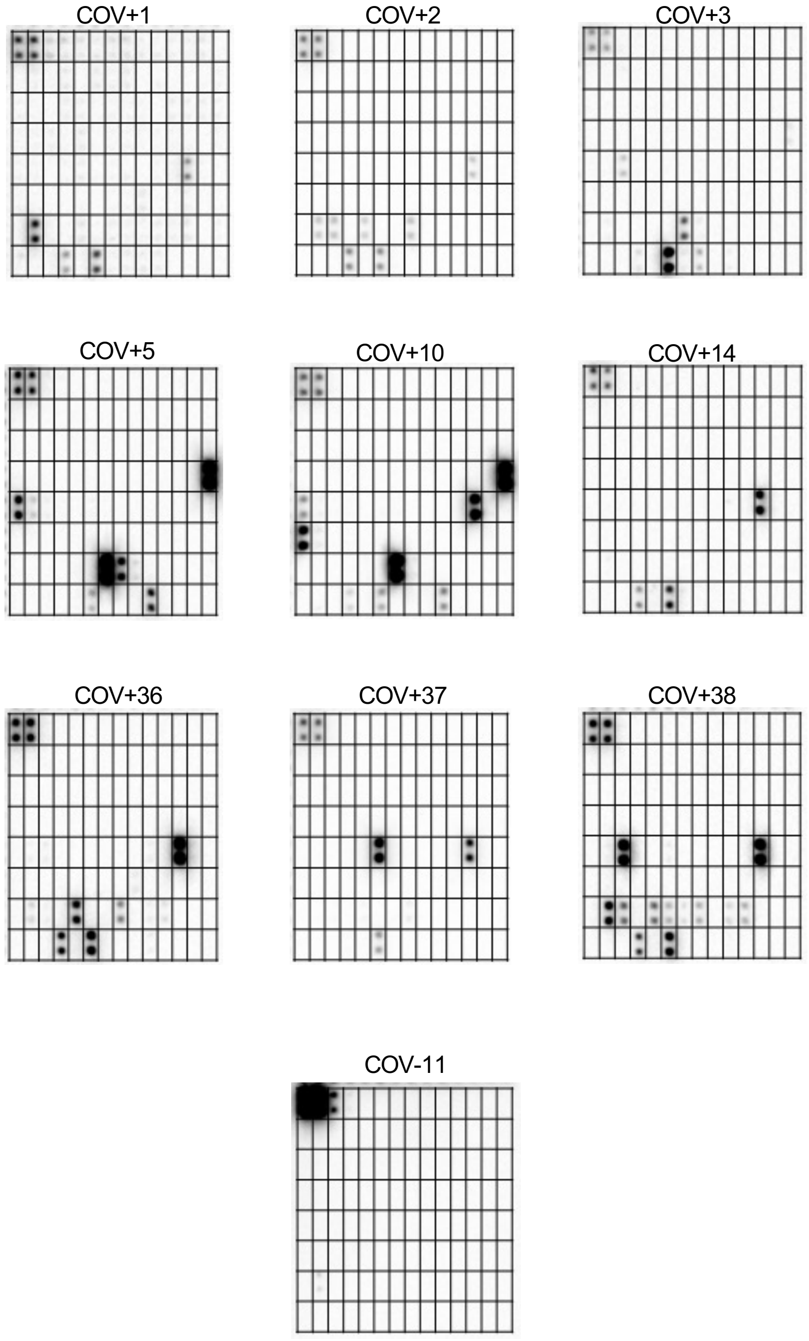

Supplemental Figure 3. Screening of a peptide array containing literature-reported epitopes. Three copies of the grid were printed on a single nitrocellulose coated glass slide. Images shown were taken with 5.2 second (COV+) or 60 second (COV-) exposure time.

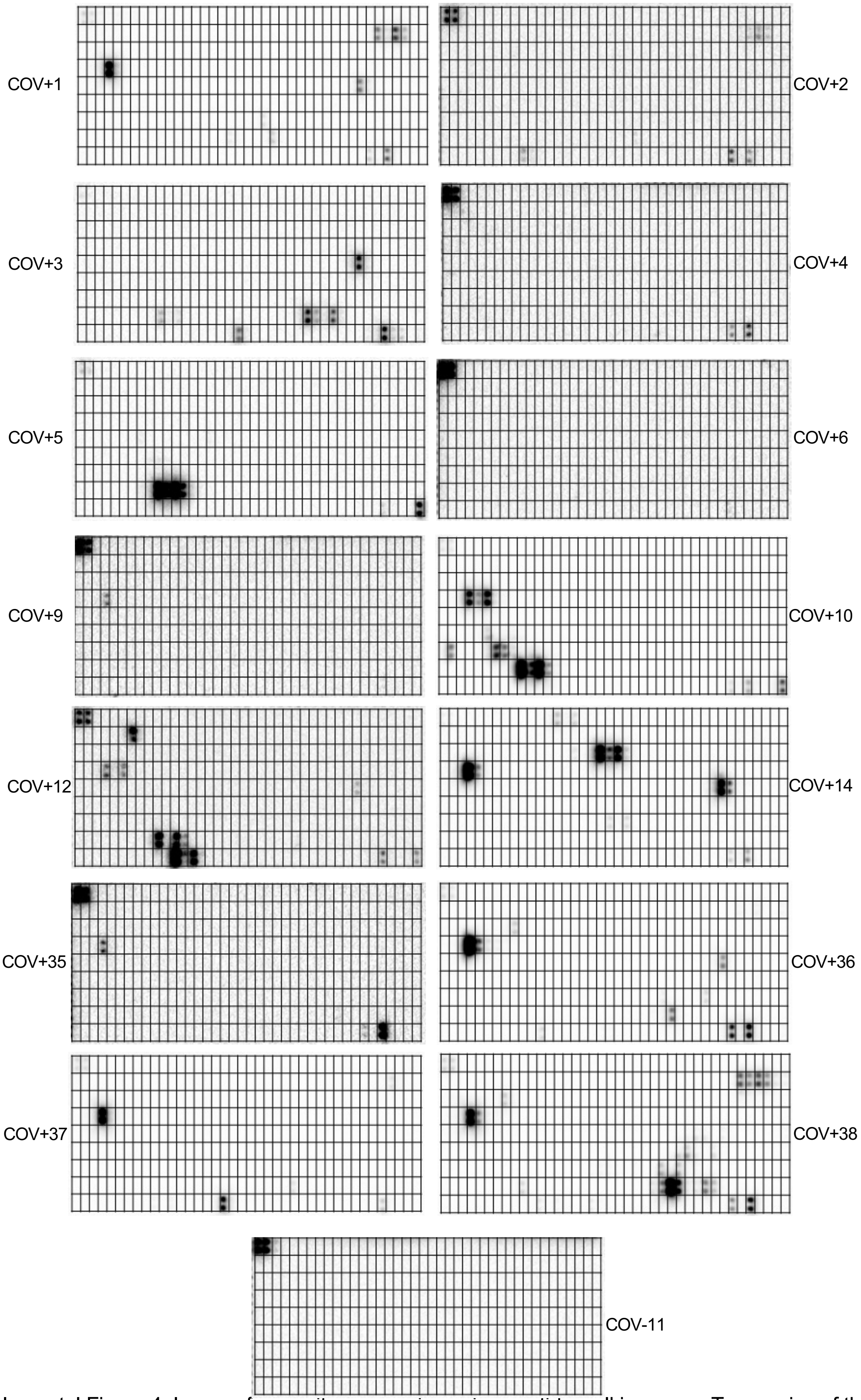

Supplemental Figure 4. Images from epitope mapping using peptide walking array. Two copies of the grid were printed on a single nitrocellulose coated glass slide. Images shown were taken with 5.2 second (COV+) or 60 second (COV-) exposure times.

A

IgG	N-6	N-156	N-221	N-246	N-361	N-381	S-21
IgG	S-176	S-451	S-551	S-671	S-811	S-881	S-1146
IgG	S-1166	S-1216	N-Dimer	N-RBD	N-FL	S-RBD	S-Ecto

B

Peptide	Sequence
N-6	PQNQRNAPRITFGGP
N-156	AIVLQLPQGTTLPKG
N-221	LLLLDRLNQLESKMS
N-246	VTKKSAAEASKKPRQ
N-361	KTFPPTEPKKDKKKK
N-381	ALPQRQKKQQTVTLL
S-21	RTQLPPAYTNSFTRG
S-176	LMDLEGKQGNFKNLR
S 451	YLYRLFRKSNLKPFE
S-551	VLTESNKKFLPFQQF
S-671	CASYQTQTNSPRRAR
S-811	KPSKRSFIEDLLFNK
S 881	TITSGWTFGAGAALQ
S-1146	DSFKEELDKYFKNHT
S-1166	LGDISGINASVVNIQ
S-1216	IWLGFIAGLIAIVMV

C

Protein	Printed Concentration
IgG	100nM
Nucleocapsid Dimerization Domain	15μM
Nucleocapsid RNA binding domain	1μM
Nucleocapsid (Full Length)	2μM
Spike-Receptor Binding Domain	14μM
Spike-Ectodomain	0.2μM

Supplemental Figure 5. SARS-CoV-2 Master Epitope Array. (A) Identity and position of peptides and proteins in the master array. All spots are printed in duplicate in the Y direction. (B) Peptide identities and sequences. (C) Protein concentrations printed on the array.

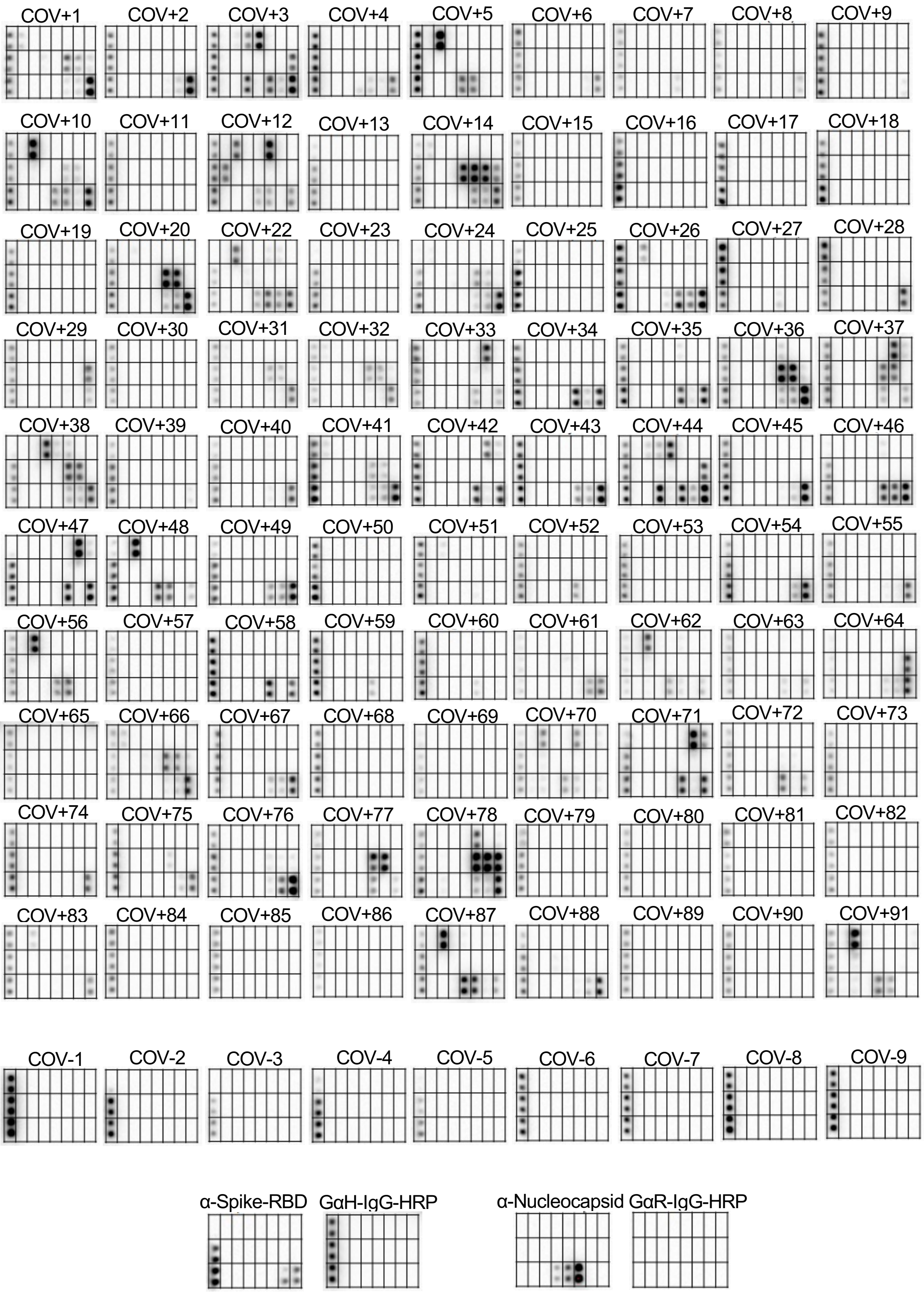

Supplemental Figure 6. Profiling antibody responses using the master array. Twenty-four copies of the grid were printed on a single nitrocellulose coated glass slide. All images shown were taken with 9.4 second exposure time.
